# Phlorofucofuroeckol A from Edible Brown Alga *Ecklonia* *Cava* Enhances Osteoblastogenesis in Bone Marrow-Derived Human Mesenchymal Stem Cells

**DOI:** 10.3390/md17100543

**Published:** 2019-09-21

**Authors:** Jung Hwan Oh, Byul-Nim Ahn, Fatih Karadeniz, Jung-Ae Kim, Jung Im Lee, Youngwan Seo, Chang-Suk Kong

**Affiliations:** 1 Marine Biotechnology Center for Pharmaceuticals and Foods, Silla University, Busan 46958, Korea; wjdghks0171@naver.com (J.H.O.); icetwig@naver.com (B.-N.A.); karadenizf@outlook.com (F.K.); jale8469@gmail.com (J.-A.K.); think3433@daum.net (J.I.L.); 2 Division of Marine Bioscience, College of Ocean Science and Technology, Korea Maritime and Ocean University, Busan 49112, Korea; ywseo@kmou.ac.kr; 3 Department of Convergence Study on the Ocean Science and Technology, Ocean Science and Technology School, Korea Maritime and Ocean University, Busan 49112, Korea; 4 Department of Food and Nutrition, College of Medical and Life Sciences, Silla University, Busan 46958, Korea

**Keywords:** alkaline phosphatase, *Ecklonia cava*, phlorofucofuroeckol A, osteoblast, huBM-MSC

## Abstract

The deterioration of bone formation is a leading cause of age-related bone disorders. Lack of bone formation is induced by decreased osteoblastogenesis. In this study, osteoblastogenesis promoting effects of algal phlorotannin, phlorofucofuroeckol A (PFF-A), were evaluated. PFF-A was isolated from brown alga *Ecklonia* *cava*. The ability of PFF-A to enhance osteoblast differentiation was observed in murine pre-osteoblast cell line MC3T3-E1 and human bone marrow-derived mesenchymal stem cells (huBM-MSCs). Proliferation and alkaline phosphatase (ALP) activity of osteoblasts during differentiation was assayed following PFF-A treatment along extracellular mineralization. In addition, effect of PFF-A on osteoblast maturation pathways such as Runx2 and Smads was analyzed. Treatment of PFF-A was able to enhance the proliferation of differentiating osteoblasts. Also, ALP activity was observed to be increased. Osteoblasts showed increased extracellular mineralization, observed by Alizarin Red staining, following PFF-A treatment. In addition, expression levels of critical proteins in osteoblastogenesis such as ALP, bone morphogenetic protein-2 (BMP-2), osteocalcin and β-catenin were stimulated after the introduction of PFF-A. In conclusion, PFF-A was suggested to be a potential natural product with osteoblastogenesis enhancing effects which can be utilized against bone-remodeling imbalances and osteoporosis-related complications.

## 1. Introduction

Prevalence of bone related complications is steadily increasing while osteoporosis and linked syndromes are causing low life quality and mortality for more of today’s elder population compared to the previous decade [1,2]. Among these complications, osteoporosis is one of the most commonly seen bone disorders and is characterized by bone formation imbalance. Bone fractures of elderly patients are mostly diagnosed with osteoporosis. Osteoporosis is being treated via different approaches, including but not limited to synthetic and natural-origin drugs which are targeting different mechanisms involved in bone formation [3,4,5]. The bone formation imbalance in osteoporosis is caused by bone volume loss while the formation of new bone tissue diminishes through inhibited osteogenesis. Hence, the stimulation of osteoblast formation is one of the main approaches to treat bone mass imbalance [6]. Additionally, links between osteogenesis and pathways related to onset of diabetes and obesity raises complex problems for elderly patients who are diagnosed with other metabolic syndromes along with osteoporosis [7,8]. Natural product research is gaining attention for the treatment or prevention of various metabolic syndromes including osteoporosis due their biocompatibility and fewer side effects compared to on the market drugs. In this context, several studies have reported the in vitro osteoblastogenesis inducing effects of marine-based nutraceuticals for the relief of osteoporotic complications [9,10].

*Ecklonia cava* (order Laminariales, family Lessoniaceae) is an edible brown alga growing abundantly on the shores of Japan and Korea where it is consumed as a part of daily diet. Numerous studies were conducted reporting its potential uses as rich source of bioactive secondary metabolites [11,12,13]. Phlorotannins are phloroglucinol oligomers [14] found richly in *E*. *cava* and credited for diverse health benefits including but not limited to antioxidant [15], antibacterial [16], anti-inflammation [17], anti-allergy [18], and anti-metastasis [19] activities. In this context, current study focused on the possible osteogenesis enhancing effect of phlorofucofuroeckol A (PFF-A) isolated from *E*. *cava*, as a part of ongoing research to elucidate natural compounds from marine sources with important bioactivities.

## 2. Results

**Figure 1:**
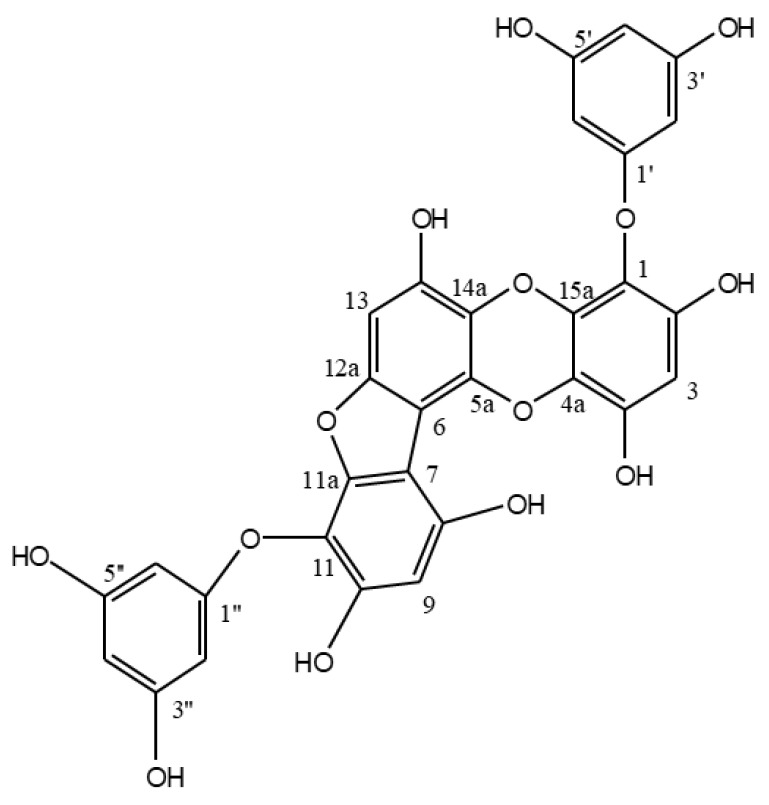
Chemical structure of phlorofucofuroeckol A (PFF-A).

PFF-A was obtained from *E. cava* and its chemical structure (Figure 1) was confirmed by comparison of ^1^H NMR and ^13^C NMR spectral data with previously published reports as described earlier [20]. PFF-A was tested for its potential activity on enhancing osteoblastic differentiation in two different cell lines; MC3T3-E1 murine pre-osteoblasts and human bone marrow-derived mesenchymal stem cells (huBM-MSCs). Cells were induced to differentiate into osteoblasts with or without PFF-A treatment to observe the enhancing effects of the PFF-A on osteoblastogenesis.

### 2.1. Effect of PFF-A on the Differentiation of Murine Pre-osteoblasts

MC3T3-E1 cells are murine calvarial osteoblast precursor cells widely used in studies for their ability to differentiate into mature osteoblasts. Therefore, any effect of PFF-A on osteoblastic differentiation was tested first in differentiating MC3T3-E1 cells. Measurement of viable cells, alkaline phosphatase (ALP) activity and extracellular mineralization as calcium deposits were used as markers for maturation into osteoblasts.

**Figure 2:**
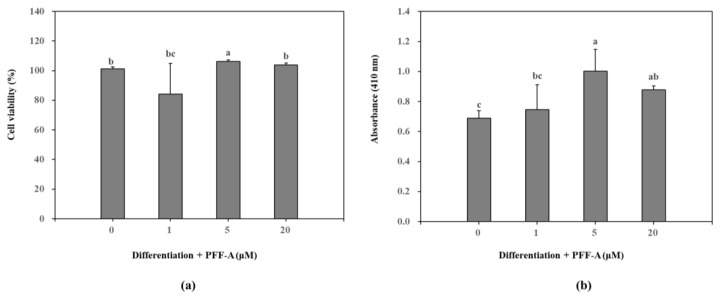
Effect of PFF-A on the cell viability (**a**) and alkaline phosphatase (ALP) activity (**b**) of MC3T3-E1 osteoblasts. Viability of cells and ALP activity were analyzed at 5 and 7 days after MC3T3-E1 cells were induced to differentiate, respectively. Cell viability was expressed as a percentage of osteoinduced untreated control group (0 μM). ALP activity was given as absorbance (410 nm) values of the colorimetric quantification of enzymatic activity. Values are means ± SD of three different experiments run in triplicate (*n* = 3). ^a–c^ Means with the different letters are significantly different (*p* < 0.05) by Duncan’s multiple range test.

**Figure 3:**
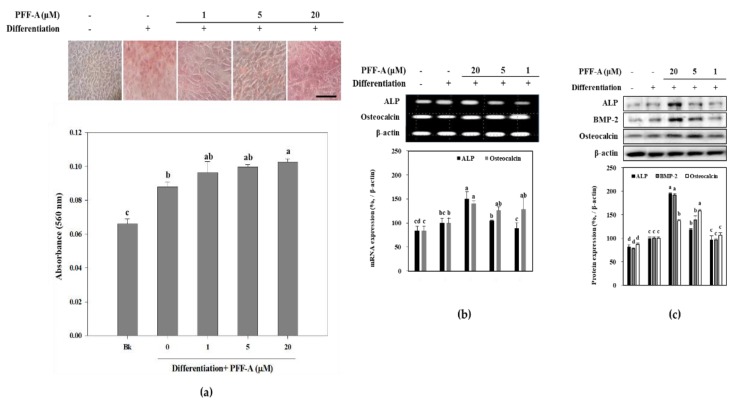
Images of MC3T3-E1 osteoblasts treated with PFF-A and stained with Alizarin Red for extracellular calcium deposits, and absorbance values (560 nm) of eluted dye retained in the cells (**a**). MC3T3-E1 osteoblasts were stained with Alizarin Red 14 days after the cells were induced to differentiate. (Bk: Non-differentiated blank group which was given growth medium instead of differentiation cocktail). Effect of PFF-A on the expression of mRNA (**b**) and protein (**c**) levels of osteoblast differentiation markers. Cells were harvested for reverse transcription-polymerase chain reaction (RT-PCR) and Western blot analysis 12 days after the cells were induced to differentiate. Expression levels quantified by densiometric analysis of bands were given as percentage of osteoinduced untreated control group after normalization using internal control β-actin. Non-induced untreated blank group was fed growth medium instead of differentiation cocktail. Values are means ± SD of three separate experiments (*n* = 3). ^a–d^ Means with the different letters are significantly different (*p* < 0.05) by Duncan’s multiple range test. Scale bar: 25 μm.

Presence of PFF-A slightly stimulated the viable cell count of MC3T3-E1 osteoblasts at 5 μM (Figure 2a) compared to the untreated control. Differentiated osteoblasts showed elevated ALP activity which was significantly enhanced after PFF-A treatment at the concentration of 5 μM (Figure 2b). Enhanced ALP activity and osteoblastogenesis was also confirmed by extracellular mineralization assessed by Alizarin Red staining. The extracellular matrix calcium deposition levels were increased during differentiation of MC3T3-E1 cells into mature osteoblasts as a marker of bone tissue formation. Treatment of MC3T3-E1 osteoblasts with PFF-A during differentiation exhibited elevated extracellular mineralization shown by quantification of the calcium staining (Figure 3a). PFF-A treatment at 5 and 20 μM resulted in enhanced calcium deposition compared to untreated control osteoblasts. Effect of PFF-A on the osteoblast differentiation pathways was analyzed by the investigation of the transcription pathways of osteoblastogenesis-related proteins. Cells treated with increasing doses of PFF-A expressed enhanced levels of ALP and osteocalcin mRNA expression (Figure 3b). In addition, protein expression levels of ALP, bone morphologic protein (BMP)-2 and osteocalcin were strongly elevated in PFF-A treated osteoblasts compared to untreated control (Figure 3c). Osteocalcin protein levels were enhanced in higher levels than its mRNA expression.

### 2.2. Effect of PFF-A on the Osteogenic Differentiation of huBM-MSCs

**Figure 4:**
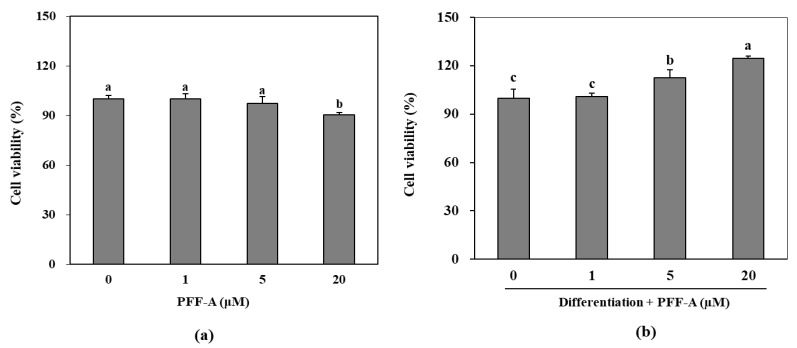
Effect of PFF-A on the viability of non-induced huBM-MSCs (**a**) and the viability of osteoinduced huBM-MSCs (**b**). Viability of osteoinduced huBM-MSCs was analyzed 5 days after inducement of differentiation. Cell viability was expressed as percentage of untreated (**a**) and osteoinduced untreated (**b**) control group (0 μM). Values are means ± SD of three separate experiment run in triplicates (*n* = 3). ^a–c^ Means with the different letters are significantly different (*p* < 0.05) by Duncan’s multiple range test.

**Figure 5:**
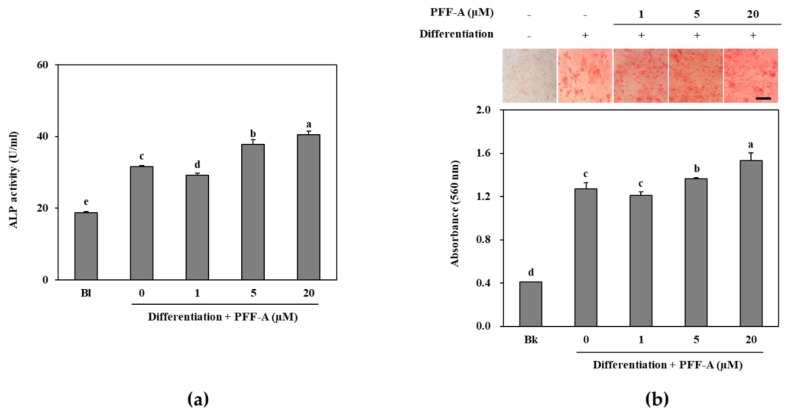
ALP activity of osteoinduced huBM-MSCs treated with PFF-A (**a**). Images of osteoinduced huBM-MSCs stained with Alizarin Red for extracellular calcium deposits and absorbance (560 nm) values of eluted dye retained in the cells (**b**). Cells were analyzed after 7 days of incubation with differentiation medium for ALP activity and 14 days of incubation with differentiation medium for Alizarin Red staining. Values are means ± SD of three separate experiments (run in triplicates for ALP activity) (*n* = 3). ^a–e^ Means with the different letters are significantly different (*p* < 0.05) by Duncan’s multiple range test (Bk: Non-differentiated untreated blank group which was given growth medium instead of differentiation cocktail). Scale bar: 50 μm.

Following the confirmation of its potential osteoblast differentiation enhancing effect in pre-osteoblasts, the mechanism behind this effect of PFF-A was investigated in bone marrow derived mesenchymal stem cells. The cytotoxicity assay showed that PFF-A treatment did not cause any significant toxicity in the huBM-MSCs up to concentration of 10 μM (Figure 4a). However, 20 μM PFF-A treatment caused a decrease in cell viability of the huBM-MSCs. Stem cells induced for osteogenic differentiation exhibited stimulated cell proliferation and PFF-A treatment (20 μM) enhanced the viable cell amount by increasing cell viability 24.53% compared to untreated control (Figure 4b). Enhancing effect of PFF-A was also confirmed on the ALP activity and extracellular mineralization of the osseous differentiated MSCs. At the highest concentration treated (20 μM), ALP activity in the osteoinduced huBM-MSCs was calculated 40.45 U/mL compared to 31.60 U/mL of untreated osteoblasts (Figure 5a). Enhancing of ALP activity was also observed as elevated extracellular mineralization. PFF-A treatment (20 μM) enhanced the extracellular calcium deposits by 20.52% compared to osseous differentiated MSCs without PFF-A treatment (Figure 5b).

**Figure 6:**
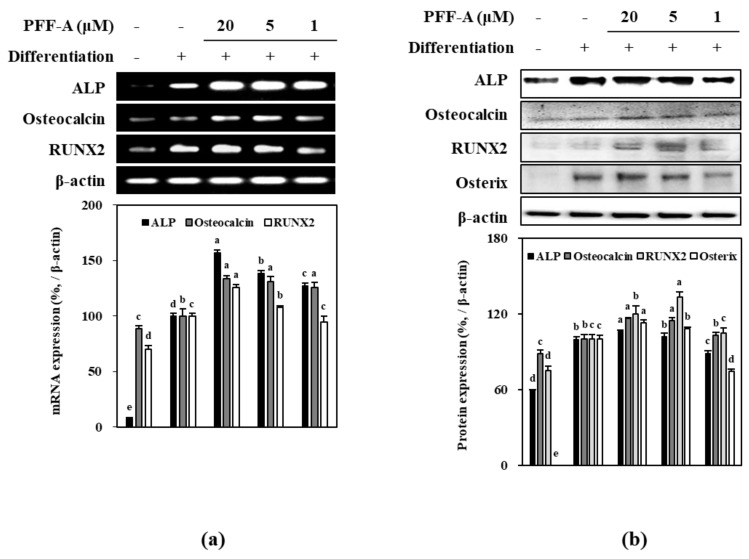
Effect of PFF-A on the expression of mRNA (**a**) and protein (**b**) levels of osteoblast differentiation markers in osteoinduced huBM-MSCs analyzed by RT-PCR and Western blotting, respectively. Cells were harvested for RT-PCR and Western blot analysis 12 days after the cells were induced to differentiate. Expression levels quantified by densiometric analysis of bands were given as percentage of osteoinduced untreated control group after normalization using internal control β-actin. Non-induced untreated blank group was fed growth medium instead of differentiation cocktail. Values are means ± SD of three separate experiments (*n* = 3). ^a–e^ Means with the different letters are significantly different (*p* < 0.05) by Duncan’s multiple range test.

Osteoblastogenesis enhancing mechanism of PFF-A was investigated by analyzing the expression of osseous differentiation inducing and regulatory pathways during the osteoinduced huBM-MSC differentiation. Osteoinduced huBM-MSCs expressed high levels of ALP mRNA as a marker of differentiation, and Runx2 and osteocalcin mRNA as a marker of osseous differentiated MSCs. Treatment with PFF-A dose-dependently increased the mRNA expression levels of ALP, osteocalcin and Runx2 compared to untreated control (Figure 6a). Consequently, protein levels of same markers (ALP, osteocalcin and Runx2) were observed to be enhanced following PFF-A treatment in a dose dependent manner (Figure 6b). In addition, osterix protein levels were investigated as osterix is a transcription factor activated by Wnt/β-catenin pathway and responsible for the osseous differentiation of huBM-MSCs. PFF-A treatment was also enhanced the levels of osterix in a dose-dependent manner (Figure 6b).

#### Effect of PFF-A on BMP and Wnt/β-Catenin Pathway

**Figure 7:**
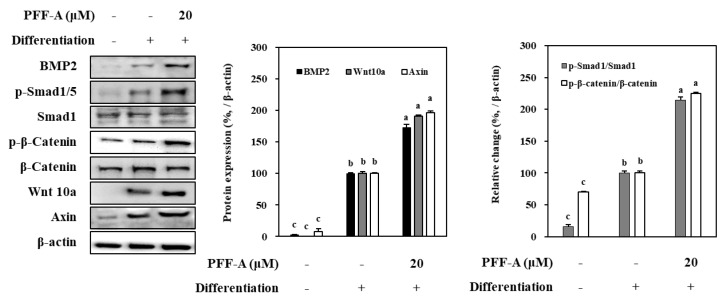
Effect of PFF-A on the levels of BMP2, Wnt 10a, and the inactive and phosphorylated (p-) Smad1 and β-catenin in osteoinduced huBM-MSCs analyzed by Western blotting. Cells were harvested for Western blot analysis 12 days after the cells were induced to differentiate. Expression levels of BMP2, Wnt 10a and Axin quantified by densiometric analysis of bands were given as percentage of osteoinduced untreated control group after normalization using internal control β-actin. Changes in p-Smad1 and p-β-catenin levels relative to their unphosphorylated forms were given as percentage of osteoinduced untreated control group after normalization using internal control β-actin. Values are means ± SD of three separate experiments (*n* = 3). ^a–c^ Means with the different letters are significantly different (*p* < 0.05) by Duncan’s multiple range test.

Mechanism of the enhancing ability of PFF-A was further analyzed by protein levels of the osteoblast differentiation ignitor pathways. Osteoinduced huBM-MSCs showed elevated levels of Smad1/5/8 complex, and β-catenin (Figure 7). Two main intracellular pathways that further activate the expression of osteoblast-specific genes. Activation of β-catenin and Smad1/5/8 complex were increased seen as the phosphorylated Smad1/5 and β-catenin were significantly higher than non-differentiated cells. Introduction of PFF-A enhanced the levels of this phosphorylation along with the expression of BMP2 and Wnt10a. However, axin, a negative regulator of the osteoblast differentiation was observed to be highly expressed during the differentiation and was not affected by PFF-A treatment (Figure 7).

## 3. Discussion

Regulation of bone remodeling is a highly critical process for a healthy bone metabolism, and any disorders of bone mass is evidently linked with variety of diseases such as arthritis, tumor growth and osteoporosis [21,22]. As the bone formation during the remodeling is dependent on the osteogenic differentiation in bone tissue, controlling the osteoblastogenesis is hence an important target to prevent or treat bone mass complications, especially osteoporosis. Bone marrow stroma derived osteoblasts are differentiated cells from mesenchymal stem cells, and they produce ALP for mineralization of the bone, and osteocalcin, which is an important hormone taking crucial roles in energy metabolism [23].

Osteoblastic differentiation of bone marrow mesenchymal stem cells is regulated by more than one signaling pathway mainly involving BMP growth factors and activation of Wnt/β-catenin/TCF cascade along with transforming growth factor (TGF)-β and mitogen-activated protein kinase (MAPK) pathways [24]. Among them, BMP pathway accompanied by β-catenin activation controls the osteoblastogenesis and subsequent formation of bone tissue by mineralization and required protein secretion.

Bone formation is carried out by increased proliferation of osteoblasts which is followed by elevated ALP activity. Results showed that PFF-A enhanced the viable cell count of osteoinduced pre-osteoblasts and huBM-MSCs. However, at 20 μM treatment PFF-A decreased the cell viability of non-induced huBM-MSCs while showing an opposite effect in osteoinduced huBM-MSCs. Although PFF-A was considered slightly cytotoxic for huBM-MSCs at 20 μM, osteoinducement was speculated to hinder or negate this effect. Increased ALP activity is a marker of osteoblast maturation and is needed for the last step of bone formation, extracellular mineralization [25]. Current data showed that PFF-A was able to increase the ALP activity in the osteoblasts differentiated from both pre-osteoblasts and stem cells. This also was confirmed by the elevated levels of osterix in huBM-MSCs treated with PFF-A. These results suggested that PFF-A had an enhancing effect on the osteogenic differentiation of cells.

Runx2 is the key transcription factor for osteoblastogenesis and has important roles in the maturation of osteoblasts. It is translocated into nucleus as a downstream factor in the Wnt and BMP pathways [26,27]. Both pathways stimulate the translocation of Runx2 and subsequent maturation of osteoblasts. Results showed that PFF-A increased the mRNA and protein expression levels of Runx2 in osteoinduced huBM-MSCs indicating that mechanism of PFF-A mediated osteoblast differentiation enhancement might occurred via enhancing the Wnt and/or BMP pathways. Therefore, it was suggested that PFF-A regulated the Wnt/β-catenin and BMP pathways which in turn induced Runx2 translocation all of which consequently elevated ALP activity and committed the stem cell differentiation to osteogenic lineage.

Mechanism of PFF-A mediated enhanced osteoblastogenesis was further analyzed via activation and expression levels of Wnt and BMP pathways. The pathway that starts with activation of BMP receptors stimulates and induces the commitment of stem cells to osteogenic lineage instead of adipogenic [28]. This signaling cascade includes phosphorylation of Smad1/5/8 complex in order to facilitate its interaction with Smad4 and their translocation to nucleus for further expression of osteoblast-specific genes via Runx2 [29,30]. Various plant-based metabolites such as flavonoids, phlorotannins, polysaccharides and similar phenol-based compounds were shown to stimulate osteoblast differentiation via BMP pathway by inducing the expression of BMP or any other activator protein in the cascade [31,32,33]. Current results showed that PFF-A presence not only increased BMP-2 expression levels for both mRNA and protein, but also notably stimulated the phosphorylation of Smad1/5/8 protein. Elevated levels of both BMP-2 and phosphorylated Smad1/5/8 compared to untreated control cells further confirmed that PFF-A-induced increase in osteoblast differentiation is related to BMP pathway.

Aside BMP pathway, Wnt/β-catenin pathway is also a key regulator for the stem cell commitment to osteogenic lineage [34]. It is heavily involved in new bone tissue remodeling and new bone formation. Activation of Wnt receptors in stem cells initiates a signaling cascade which involves activation and translocation to nucleus of β-catenin and stimulates the transcription of osteoblast-specific genes such as ALP and osterix [26]. The results showed that huBM-MSCs treated with PFF-A exhibited increased levels of Wnt-10a and phosphorylation of β-catenin. Significant increase in the activation ratio of β-catenin clearly suggested that Wnt/β-catenin pathway was also involved in the enhancing stem cell osteoblastogenesis by PFF-A treatment.

Although there are not any reports on specific side-effects of PFF-A, it could be speculated that it shares the common disadvantages of phlorotannins. Phlorotannins are known to be non-specific inhibitors of several enzymes that act in digestive track such as α-glucosidase and α-amylase [35,36]. Also, it was reported that tannin derivatives could cause irritation in stomach and potential damage in liver [37]. However, the doses that shown to exert serious side effects are significantly higher and not comparable to the tested concentrations. Although, PFF-A was assumed to be possess minimal side effects further studies to evaluate its dosage and relative side-effects would be urged.

In conclusion, present study showed that PFF-A could enhance the osteogenic differentiation of pre-osteoblast and huBM-MSCs. Results demonstrated that PFF-A stimulated the osteoblastogenesis by interacting with BMP and Wnt/β-catenin pathways and consequently by increasing ALP activity and mineralization. As a result, PFF-A was suggested to be potential natural compound with therapeutic properties against osteoporosis and related bone complications that urged further studies to elucidate its detailed action mechanism and bioavailability.

## 4. Materials and Methods

### 4.1. Isolation of PFF-A from Plant Material

*E. cava* was collected between October 2014 and March 2015 from seashores of Jeju Island, Korea. Specimens were identified by the corresponding author and a voucher sample was stored. Fronds of *E. cava* were freeze-dried and stored at 25°C prior to experiments. Freeze-dried samples were ground to powder (4.0 kg) and extracted three times using EtOH (3 × 10 L). Solvent-partition of the crude extracts (584.3 g) was carried out with n-hexane, n-BuOH and 85% aq. MeOH to yield solvent fractions along with H_2_O residue. Forty grams of the n-BuOH fraction was further separated by a silica gel column chromatography (60, 0/063–0.200 mm) (Merck, Kenilworth, NJ, USA) with the gradient mixture (30:1 to 1:1) of CH_2_Cl_2_:MeOH and CHCl_3_:MeOH, respectively. Phlorofucofuroeckol A (52.2 mg) was isolated as a light brown powder from 3:1 CHCl_3_:MeOH sub-fraction by high performance liquid chromatography (HPLC) (C18, 40% aq. MeOH). The chemical structure of phlorofucofuroeckol A (Figure 1) was verified by spectral data as previously reported [20].

### 4.2. Cell Culture and Differentiation

Murine osteoblast-like MC3T3-E1 cells obtained from ATCC (CRL-2593^™^) and huBM-MSCs obtained from PromoCell (C-12974) were cultured in 6-well plates unless otherwise noted. MC3T3-E1 cells were fed with α-Modified minimal essential medium (αMEM) containing 10% fetal bovine serum (heat-inactivated, *v*/*v*), 1 mM sodium pyruvate, 100 units/L penicillin and 100 mg/L streptomycin in an atmosphere of 5% CO_2_ at 37 °C. Following the confluence, osteoblast differentiation was induced with a differentiation cocktail of 50 μg/mL ascorbic acid and 10 mM β-glycerophosphate in cell culture medium. PFF-A was introduced to the cells with differentiation medium and included in every medium change (every second day).

huBM-MSCs were fed with mesenchymal stem cell growth medium (C-28009, PromoCell) and incubated in an atmosphere of 5% CO_2_ at 37 °C. Following 100% confluence cells were induced by MSC osteogenic differentiation medium (C-28013, PromoCell) and incubated until the time of analysis. PFF-A was introduced with differentiation medium and included in all medium changes (every third day).

### 4.3. Cell Viability Assay

Effect of PFF-A on the viability of MC3T3-E1 osteoblasts, and non-induced and osteoinduced huBM-MSCs was evaluated by 3-(4,5-dimethylthiazol-2-yl)-2,5-diphenyltetrazolium bromide (MTT) assay as previously described [20]. Viability of MC3T3-E1 cells were measured at the day 5 of differentiation inducement. Viability of non-induced huBM-MSCs that fed growth medium only was measured after 2 days of incubation with or without PFF-A treatment while the viability of osteoinduced huBM-MSCs was analyzed at the day 5 of differentiation. PFF-A was introduced to the cells at the beginning of incubation or differentiation inducement and included in all medium changes until the day of analysis (every second day for MC3T3-E1 and every third day for huBM-MSC). Following the treatment period of the cells with PFF-A, cell culture medium was aspired, and wells were added 100 μL of MTT reagent (1 mg/mL) prior to 4 h incubation. Formazan salt was dissolved with the addition of 100 μL DMSO to the wells. Viability and proliferation of the cells were calculated by measuring the absorbance values of the formed formazan salts for each well at 540 nm using a Multiskan GO microplate reader (Tecan Austria GmbH, Grodig, Austria) and plotting the values as a percentage of untreated control cells.

### 4.4. Cellular Alkaline Phosphatase (ALP) Activity

ALP activity was evaluated in differentiated MC3T3-E1 and osteoinduced huBM-MSCs treated with or without PFF-A. Differentiated MC3T3-E1 osteoblasts were used for ALP activity assay at day 7 of differentiation. Also, a blank group which was not induced and untreated was analyzed after 7 days of incubation in growth medium. Cells were washed with phosphate buffer saline (PBS) and lysed with 0.1% Triton X-100 and 25 mM carbonate buffer. The cellular ALP activity was assessed using the supernatants of the cell lysates following centrifugation at 4 °C 12,000× *g* for 15 min. Total protein contents of the supernatants were analyzed and normalized by the Bradford protein determination method. The absorbance of the reactive solution containing the supernatants and the enzyme reaction buffer (15 mM p-nitrophenyl phosphate, 1.5 mM MgCl_2_ and 200 mM carbonate buffer) was measured at 405 nm after 15 min. of incubation, using a Multiskan GO microplate reader (Tecan Austria GmbH, Grodig, Austria).

Osteoinduced huBM-MSCs were analyzed for ALP activity using a commercial kit following the manufacturer’s instructions. Cells were prepared for ALP activity assay at day 7 of treatment and the data was obtained as absorbance values at 560 nm using a Multiskan GO microplate reader (Tecan Austria GmbH). Also, a non-induced huBM-MSC blank group was analyzed after 7 days of incubation with growth medium instead of differentiation medium.

### 4.5. Alizarin Red Staining

Extracellular calcium deposits of differentiated MC3T3-E1 and osteoinduced huBM-MSCs were investigated and quantified by Alizarin Red staining with or without PFF-A treatment at day 14 of differentiation. Also, a non-induced untreated blank group which was fed growth medium instead of differentiation medium was stained for calcium deposits after 14 days of incubation. Cells were fixed on 6-well plates with 30 min incubation in 70% ice-cold ethanol which was followed by removal of ethanol and washing with distilled H_2_O. Staining was carried out by introduction of Alizarin Red solution followed by 10 min incubation at room temperature. After the incubation, the Alizarin Red solution was aspired from wells and cells were washed with distilled H_2_O to remove to unbound Alizarin red stain. Stained calcium deposit images showing extracellular mineralization were taken by an Olympus microscope (Tokyo, Japan). Subsequently, the Alizarin red dye was eluted from wells with 10% cetylpyridinium chloride (Sigma-Aldrich, St. Louis, MO, USA) solution and mineralization was quantified by absorbance values at 560 nm using a Multiskan GO microplate reader (Tecan Austria GmbH).

### 4.6. Reverse Transcription-Polymerase Chain Reaction Analysis

Total RNA was obtained from differentiated MC3T3-E1 and osteoinduced huBM-MSCs with or without PFF-A treatment using Trizol reagent (Invitrogen, CA, USA) at day 12 of differentiation. Also, a non-induced untreated blank group which was fed growth medium instead of differentiation medium was analyzed after 12 days of incubation. Synthesis of cDNA was started with addition of total RNA (2 μg) to RNase-free water containing oligo (dT) and followed by denaturation at 70 °C for 5 min. Next, the mixture was reverse transcribed in a master mix (1 X RT buffer, 1 mM dNTPs, 500 ng oligo (dT), 140 U M-MLV reserve transcriptase and 40 U RNase inhibitor) using an automatic T100 Thermal Cycler (Bio-Rad, Hercules, CA, USA) with a cycle of 42 °C for 60 min and 72 °C for 5 min. Sense and antisense primers previously detailed [38] were used for the amplification of the target cDNA. The cDNA amplification was carried out using T100 Thermal Cycler (Bio-Rad) with cycle settings at 95 °C for 45 s, 60 °C for 1 min and 72 °C for 45 s for 30 cycles Final PCR products were separated by gel electrophoresis on 1.5% agarose gel for 30 min at 100 V. Bands were then observed following the staining with 1 mg/*mL* ethidium bromide under UV light using CAS-400SM Davinch-Chemi imager^TM^ (Seoul, Korea).

### 4.7. Western Blotting

Protein immunoblotting was performed with standard Western blotting procedures. Differentiated MC3T3-E1 and osteoinduced huBM-MSCs with or without PFF-A treatment were lysed by pipetting with 1 mL of RIPA lysis buffer (Sigma–Aldrich) at 4 °C for 30 min at day 12 of differentiation. Also, a non-induced untreated blank group which was fed growth medium instead of differentiation medium was analyzed after 12 days of incubation. The acquired cell lysate (25 μg) was used for Western blot analysis. Proteins in cell lysate were separated by sodium dodecyl sulphate-polyacrylamide gel electrophoresis (SDS-PAGE) on 4% stacking and 12% separating gels. Proteins subjected to separation were then electrotransferred to a polyvinylidene fluoride membrane (Amersham Biosciences, Little Chalfont, England, UK), which was blocked with 5% skim milk powder in TBST buffer after transfer. The membrane was hybridized with primary antibodies diluted (1:1000) in primary antibody dilution buffer containing 1X TBST with 5% bovine serum albumin at 4 °C overnight and incubated with horseradish-peroxidase-conjugated secondary antibody at room temperature for 2 h. Immunoreactive proteins bands were visualized by a luminol-based chemiluminescence assay kit (Amersham Biosciences) according to the manufacturer’s manual. Images of protein bands were captured using a Davinch-Chemi imager^TM^ (CAS-400SM, Seoul, Korea).

### 4.8. Statistical Analysis

The data were presented as mean of three independent experiments ± SD. Statistically significant differences among the means of the individual test groups were determined by one-way analysis of variance (ANOVA) followed by Duncan’s multiple range tests using SAS v9.1 software (SAS Institute, Cary, NC, USA), *p* < 0.05 being the defining level for the significance of differences.
